# Genetic variation and evolutionary history of a mycorrhizal fungus regulate the currency of exchange in symbiosis with the food security crop cassava

**DOI:** 10.1038/s41396-020-0606-6

**Published:** 2020-02-17

**Authors:** Romain Savary, Cindy Dupuis, Frédéric G. Masclaux, Ivan D. Mateus, Edward C. Rojas, Ian R. Sanders

**Affiliations:** 1 Department of Ecology and Evolution, University of Lausanne, 1015 Lausanne, Switzerland; 2 Laboratory for Biological Geochemistry, School of Architecture, Civil and Environmental Engineering, École Polytechnique Fédérale de Lausanne, Lausanne, Switzerland; 3 Vital-IT Group, Swiss Institute of Bioinformatics, University of Lausanne, 1015 Lausanne, Switzerland; 4 Department of Plant and Environmental Sciences, University of Copenhagen, 1871 Copenhagen, Denmark

**Keywords:** Microbial ecology, Fungal ecology

## Abstract

Most land plants form symbioses with arbuscular mycorrhizal fungi (AMF). Diversity of AMF increases plant community productivity and plant diversity. For decades, it was known that plants trade carbohydrates for phosphate with their fungal symbionts. However, recent studies show that plant-derived lipids probably represent the most essential currency of exchange. Understanding the regulation of plant genes involved in the currency of exchange is crucial to understanding stability of this mutualism. Plants encounter many different AMF genotypes that vary greatly in the benefit they confer to plants. Yet the role that fungal genetic variation plays in the regulation of this currency has not received much attention. We used a high-resolution phylogeny of one AMF species (*Rhizophagus irregularis*) to show that fungal genetic variation drives the regulation of the plant fatty acid pathway in cassava (*Manihot esculenta*); a pathway regulating one of the essential currencies of trade in the symbiosis. The regulation of this pathway was explained by clearly defined patterns of fungal genome-wide variation representing the precise fungal evolutionary history. This represents the first demonstrated link between the genetics of AMF and reprogramming of an essential plant pathway regulating the currency of exchange in the symbiosis. The transcription factor RAM1 was also revealed as the dominant gene in the fatty acid plant gene co-expression network. Our study highlights the crucial role of variation in fungal genomes in the trade of resources in this important symbiosis and also opens the door to discovering characteristics of AMF genomes responsible for interactions between AMF and cassava that will lead to optimal cassava growth.

## Introduction

The majority of terrestrial plant species is colonized by arbuscular mycorrhizal fungi (AMF) forming the mycorrhizal symbiosis [1]. There is great interest in this mutualistic symbiosis both from an ecological and agronomic perspective because the fungi significantly improve plant growth [2]. All mutualisms involve the trading of currencies conferring fitness benefits, as well as representing a potential cost. Consequently, both partners are expected to evolve regulatory mechanisms to prevent overexploitation by the partner [3]. While genes crucial to the currency of trade have been identified for many mutualists, the demonstration that genetics of one partner plays a role in the variation in obtention of an essential currency in the other partner has not been the focus of much research.

A common method used to explore genes essential to the symbiosis between plants and AMF is to genetically dissect the plant [4], mostly by looking at plant mutants able, or only partially able, to form the symbiosis. This approach has not been possible with the fungal partner [5], primarily because of the lack of a gene knockout or transformation system, and has resulted in a strong bias towards knowledge of plant genes involved in the symbiosis. It has also given the appearance that the plant has control over the fungal symbiont because it only results in the discovery of plant genes that do not allow normal development of the fungus in the first days of symbiosis establishment [6] rather than genes involved in a functioning mutualistic symbiosis following establishment. As the genetic dissection of the fungal partner is not currently feasible, the only possibility to observe the effect of the fungus on the plant is to minimize the effect of plant variation, by using a single clonal species of plant and apply a wide array of genetically different AMF isolates.

Recent ground-breaking research on the mycorrhizal symbiosis has revealed that plant genes control whether the fungus receives one essential currency in this trade, namely lipids [7–10]. Several lines of evidence led to the discovery of the importance of plant-derived lipids for the fungi [7]. The discovery of the lack of fatty acid synthases (FAS) genes in AM fungi suggested the inability of AMF to produce their own fatty acids [11, 12]. At the same time, only plant species engaging in AMF symbiosis possess a specific and central gene of the fatty acid synthesis, namely the acyl-ACP thio-esterase *FatM* [13]. *FatM* in combination with three other genes conserved in mycorrhizal plants; RAM2 and the ABC transporters STR/STR2 were suggested to be the essential modules for the final synthesis of potential 16:0 β-monoacylglycerol, which are then potentially transported in the fungus [14]. Another line of evidence is the change in lipid content when the plant enters into symbiosis [11]. Moreover, the genes involved in the fatty acid synthesis pathway in plants are switched on during colonization by the fungus [7]. However, studies typically only use one isolate of the fungus, usually the model isolate of *Rhizophagus irregularis* (DAOM197198, [7, 15]). While such approaches show the existence of an “on–off switch” of these crucial plant genes, they ignore the role that variation in the fungus plays in the regulation of this currency exchange, and yet understanding regulation of the currencies of trade is essential to understand the stability of mutualisms. Understanding the variation in molecular regulation of symbiosis in important crop plants in response to a natural diversity of AMF is crucial for future field applications. This is particularly true for the interaction between *R. irregularis* and the food security crop cassava (*Manihot esculenta*). Almost one billion people eat cassava daily and the association between this fungus and cassava has been shown to result in significantly higher productivity in conventional cassava farming [16, 17].

In nature, plants do not just encounter one fungus but many different mycorrhizal fungi, of different species, and different genotypes of the same species. Ecological studies demonstrate the strong role of AMF intraspecific genetic variation in variation of plant growth responses [18–20]. This suggests that plant molecular regulation of trade in the symbiosis could vary strongly in response to different AMF isolates of the same species. The physiological interaction in the symbiosis is known to be bipartite, involving bidirectional exchange of essential resources that represent the currency of this trade [3]. Given that evolutionary theory suggests that mechanisms to regulate trade should exist in both partners to combat overexploitation [3], and that genetically different fungi of the same species vary greatly in the benefit they confer to plants, we hypothesized that genetic variation within one AMF species impacts the plant transcriptional regulation of a currency involved in trade in the symbiosis.

The study involved two steps. Firstly, it was necessary to demonstrate that, indeed, the lipid biosynthesis pathway in cassava is commonly upregulated by many genetically different AMF as previous studies have concentrated on so few different fungi. Secondly, we tested whether clearly discernible patterns of genetic variation within a single AMF species, and reflecting their evolutionary history, influence the regulation of a plant gene pathway that is considered fundamentally important in trade between the two partners of the symbiosis; namely the pathway from initiation to fatty acid synthesis [7–11]. Until recently, it was not possible to test this because of the lack of a very high-resolution phylogeny showing the genetic relationships within a single AMF species.

**Fig. 1:**
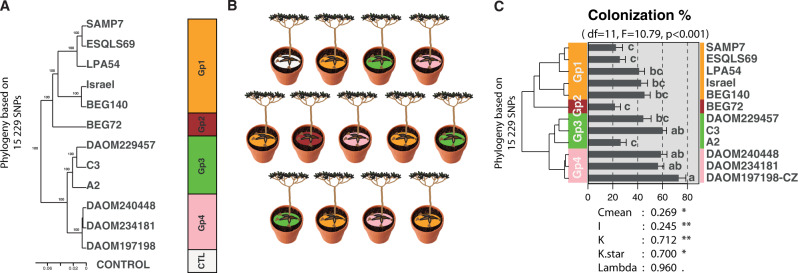
Experimental design. **a** Phylogeny of the 12 isolates of *R. irregularis* based on 15 229 SNPs generated from ddRADseq [21], and used as inoculation treatments. **b** Experimental design of one block comprising one replicate of every randomized inoculation treatment. Each block was replicated 16 times. **c** AMF colonization and its association with the fungal ddRADseq phylogeny. Different letters next to bars indicate a significant difference (*P* *<* 0.05). Five phylogenetic signal indicators (Cmean, I, K, KStar, Lambda) are displayed with the significance of the test for an association between percentage root length colonization and the phylogeny (*P* < 0.1, *P < 0.05, **P < 0.01) which are shown below in the graph. Color coding for each of the four genetic groups follows [21] (GP1: orange, GP2: brown, GP3: green, GP4: pink).

We capitalized on the recently published high-resolution ddRADseq data of the AMF *R. irregularis* [21] in order to build a new high-resolution phylogeny based on 15229 genome-wide SNPs, 100% shared across all isolates. Twelve *R. irregularis* isolates, representing the four genetic groups (Gp1, Gp2, Gp3, Gp4) of *R. irregularis* were chosen (Fig. 1a) [21]. We inoculated cassava (cultivar NGA16) with each of the 12 isolates (Fig. 1b). All plants were micro-propagated clonally, eliminating plant genetic variability and permitting us to clearly define the effects of fungal variation on plant gene transcription. All fungi have been subcultured for many years in identical in vitro conditions to remove environmental effects that could have been due to isolates originating from a heterogeneous environment. We sequenced the cassava root–fungal transcriptome after the partners had formed symbioses for several months and retrieved both plant and fungal transcripts. This experimental approach is unique, employing a design with phylogenetically defined fungal isolates, coupled with dual RNA sequencing of the plant and fungus in symbiosis. We found that fatty acid synthesis in cassava is strongly activated by all *R. irregularis* isolates. Moreover, the variation in the expression of fatty acid synthesis genes was associated with patterns of *R. irregularis* genetic variation and evolutionary history. Because strong variation in plant gene transcription was generated in response to fungal genetic variation, we were also able to build networks of plant co-expressed genes in order to detect hub genes central to this important metabolic pathway that is upregulated in symbiosis. With this method we identified one fatty acid plant co-expression network dominated by the transcription factor RAM1 and coupled with several dominant fatty acid genes and other transcription factors.

## Material and methods

### Fungal material

Twelve isolates of *R. irregularis* [21] were grown with Ri T-DNA transformed carrot roots in in vitro culture for a period of three and half months [22]. The isolates spanned the phylogeny of this species and represented the four *R. irregularis* genetic groups described in [21]. The isolates representing the four groups were SAMP7, ESQLS69, LPA54, BEG140, and Israel (Gp1), BEG72 (GP2), C3, DAOM229457, and A2 (Gp3), and DAOM243181, DAOM240448, and DAOM197198-CZ (Gp4; Fig. 1a). All isolates were maintained in identical in vitro conditions to reduce environmental effects. Details of isolate origin are found in [21].

### Plant material and experimental design

The *Manihot esculenta* cultivar NGA16 was obtained from the International Centre for Tropical Agriculture (https://ciat.cgiar.org/). It was micropropagated clonally. Following micropropagation [23], plantlets were grown individually in glass tubes in M1 phytagel with 14 h of daylight (light intensity 100 μE m^−2^ s^−1^) at 25 °C in a growth chamber. After 30 days, noncontaminated plantlets were placed in 0.37 L pots in a steam-sterilized soil (105 °C for three consecutive sessions of 20 min) comprising Seedling substrate (Klasmann) and perlite (1:1) on tables in the greenhouse with constant conditions (28 °C, 70% RH and 16 h daylight). Young plants were protected from full light with a mesh for 37 days of acclimation. The plants were transferred to 2 L pots with a newly sterilized substrate composed of substrate S4 (Klasmann), perlite, quartz sand, and clay (1:1:1:1) in the greenhouse with the same conditions for 30 days before inoculation.

### Design

Two hundred and eight plantlets were randomly chosen for the experiment. Thirteen plantlets were assigned for inoculation with one of the 12 fungal isolates or assigned as mock-inoculated controls (CTL) (Fig. 1a, b). Plants were inoculated with 300 spores of one of the twelve isolates, suspended in 10 ml of pure ddH_2_O. CTL plants were inoculated with 10 ml of pure ddH_2_O without AMF. Inoculation was carried out on sixteen clones of NGA16 for each fungal treatment and the CTL. Two replicates per treatment were randomly assigned to one of the eight blocks and to one position within the block. Thus, each block contained all the treatments arranged randomly (Fig. 1b). The blocks were rotated every two weeks in order to avoid microclimate effects.

### Measurements

Following 120 days of growth, plants were harvested by block. Root samples were collected in less than one minute per sample. Three samples of fine roots i.e., non-starchy roots, typically around ten pieces of roots from different depths, were collected and kept in liquid nitrogen before being stored at −80 °C. Another sample of non-bulking roots was kept at −20 °C for estimation of intraradical fungal colonization (Supplementary Note S1). Plants were dried in a drying oven for 72 h at 60 °C and then the above ground dry mass (ADM), the total root dry mass (RDM) and the bulking-root dry mass (BDM) were measured (Data S1).

### RNA library preparation and sequencing

RNA was extracted from 47 root samples (Data S2). RNA from 40 samples was extracted following a previously published protocol [24]. The remaining seven samples (Data S2), were extracted with the Maxwell^TM^ 16 robot (Promega) with the Maxwell^®^ 16 LEV Plant RNA Kit following the manufacturers instructions. Because of cost we could only extract and sequence RNA from 3–4 replicates per treatment of the 16 replicates. RNA from nine replicates of the CTL treatment was extracted and sequenced. Concentration and integrity of the RNA samples were assessed with a Nanodrop 2000 and a Fragment Analyser^TM^ (Advanced Analytical), using the  RNA quality number (RQN) integrity score.

Libraries were constructed by polyA selection of RNA. Each library was prepared with the TruSeq Stranded mRNA Sample Prep Kit^®^ (Illumina). Library concentration was assessed with Quantifluor (Promega) and quality with a Fragment Analyser^TM^ (Advanced Analytical). Libraries were sequenced using Illumina HiSeq2000 paired-end (2 × 100 nt). More details on extraction and sequencing can be found in Supplementary Note S2.

### Statistical analysis of cassava quantitative traits

The ADM, RDM, BDM, and AMF colonization of each *M. esculenta* plant was analyzed using one-way ANOVA and significant differences between means were assessed with a post hoc Tukey HSD test. To measure the AMF phylogenetic signal in plant quantitative growth traits, a phylogenetic tree was built using the variation among the deepest ddRADseq sequenced replicate of each of the 12 isolates [21, 25]. We measured scalar distances [25] using the variation found in the genome among all samples. This variation was based on shared markers in all isolates of 15,229 SNPs, 1085 insertions, 1455 deletions, and 14 multiple-nucleotide polymorphisms. This tree was used to calculate whether a significant phylogenetic signal existed between quantitative traits of cassava plants and the *R. irregularis* phylogeny, using the phylosignal package [26]. The phylogenetic signal was measured on the four variables using five types of phylogenetic signal indicators: Moran’s I index [27], Abouheif’s Cmean index [28], Blomberg’s K and K* [29], and finally Pagel’s λ [30].

### Bioinformatic pipeline

The quality of paired-end reads in all 47 libraries was first checked with FastQC [31]. Illumina adapters were removed from each library. Low-quality nucleotides or reads smaller than 40 bp were removed with Trimmomatic version: 0.33 [32]. A 4-base wide sliding window was applied in order to cut sections of reads with an average quality lower than a Phred score of 15.

The data were pseudo-aligned with Kallisto version 0.42.4 [33] to obtain estimate counts based on an index of transcripts from both *M. esculenta* v6.1 transcripts [34] and on *R. irregularis* predicted genes. The gene prediction was made using a new annotation of the *R. irregularis* DAOM197198 single nucleus genome assembly N6 [35]. For details of the new annotation see Supplementary Note S3. The *tximport* function [36] was used to create a data table of estimated counts obtained from Kallisto in the R environment (www.CRAN.R-project.org; R Development Core Team 2008).

A second strategy was applied by mapping the reads with the 2-pass aligner STAR version 2.5.1b [37] on both the *M. esculenta* genome assembly v6.1 [34] and the N6 *R. irregularis* single nucleus genome assembly [35]. Raw counts were then obtained from the .bam file using the command *featureCounts* from the Rsubread package [38] with the *M. esculenta* v6.1 annotation file and a new annotation file based on the new gene prediction of the N6 *R. irregularis* genome.

The four count tables (two from Kallisto and two from STAR and *featurecounts*), containing the counts of the 47 libraries, were then analyzed in parallel. Global visualization of both the *M. esculenta* and *R. irregularis* data was obtained by normalizing the Kallisto estimated counts with variance stabilization transformations applied through the *varianceStabilizingTransformation* function and then by using the plotPCA function in DESeq2 [39]. Differential analysis was run with the DESeq2 package [39] using transcripts in *M. esculenta* and genes in *R. irregularis*. Differentially transcribed (DT) *M. esculenta* transcripts and *R. irregularis* genes were only retained if present in the DESeq results following both methods of mapping and read counting (Kallisto and 2passStar-*featureCounts*). However, for simplicity only the results from Kallisto were used for representation in the figures. Details of the differential transcription analysis for *M. esculenta* and *R. irregularis* can be found in Supplementary Note S4. All mapping information is provided in Data S3a–e.

### Gene ontology (GO) enrichment analysis

The GOseq package [40] was used with all DT gene lists to perform GO enrichment analysis in accounting for gene length bias. A false discovery rate was applied in the detection of enriched GO terms [41].

### Testing for an association between cassava fatty acid gene transcription and the fungal phylogeny

A test of significant phylogenetic conservatism of cassava fatty acid gene transcription across the fungal phylogeny would suggest that transcription of these plant genes is driven by the evolutionary history of this fungal species. We tested this relationship with cassava genes that were commonly DT in response to inoculation with *R. irregularis*. We calculated the five different phylogenetic signal indices and *P* value of their respective test [26]. These five indices represent a statistic used to calculate the probability of an association between a phylogenetic pattern and a given quantitative trait. We used the same phylogenetic tree used to measure the phylogenetic signal on plant quantitative traits.

### Cassava orthologs of fatty acid genes

Genes related to the fatty acid pathway described in other species, mainly *Medicago truncatula*, were retrieved as proteins and were blasted with blastp [42] against *M. esculenta* proteins. Orthologs in the cassava dataset with high similarity to *M. truncatula* fatty acid proteins were retained. Neighbor-joining trees were built on the basis of protein sequences of genes involved in the fatty acid pathway using MEGA7 [43]. The protein sequences of the fatty acid genes in the original species were retrieved from NCBI.

### Co-transcription analysis

We performed gene co-expression analysis with the package weighted correlation network analysis (WGCNA, [44]). Such analysis has the advantage over differential transcription analyses in that it can detect genes that potentially did not change significantly in mean transcription between two conditions but that are co-transcribed across a wide range of conditions and that are central in the co-regulation network by highly correlating with a large number of other genes. This is only possible to detect in a complex dataset where experimental treatments resulted in changes in gene transcription. Such an analysis would not be possible in simple mycorrhizal versus mock-inoculated experimental design. We use this method to (i) generate cassava gene modules, and (ii) to generate “symbiosis” gene modules by combining plant and fungal gene transcription.

We used the counts obtained from Kallisto of both species. We removed CTL libraries, as well as the low count genes with a minimum of 1 read per library, and we removed low variation genes. The counts were normalized using the *varianceStabilizingTransformation* function of DESeq2 as suggested by Langfelder and Horvath (FAQ, WGCNA website, 2014). Libraries that were outliers were removed based on hierarchical clustering (A2–13, ESQLS69–10, ESQLS69–13, DAOM234181–7).

We performed the analysis in two ways. First, we implemented a simple gene module analysis of *M. esculenta* gene transcription. We then identified the cassava gene module containing the RAM1 gene. Second, we repeated this analysis, this time merging all the cassava and fungal gene transcripts in order to generate “symbiosis gene modules” that can potentially contain co-expressed plant and fungal transcripts. We then searched for the module containing RAM1. With WGCNA, we also calculated module membership (MM), a value of connectivity of each gene in the network. Genes with an MM value above 0.9 are considered as hub genes because of their high connectivity to other genes in the module. Hub genes in these modules were then inspected manually. In both modules, we identified the function of the different genes and we used GOSeq to perform GO enrichment analysis on the hub gene lists and the total gene list. Visualization of the Cyan-RAM1 module network was performed with Cytoscape [45] and properties of the network were calculated with the NetworkAnalyzer option of Cytoscape. The degree of each node and the weight of each edge were used to represent the network.

## Results and discussion

### Patterns of *R. irregularis* transcriptome variation are congruent with patterns of *R. irregularis* genome variation

**Fig. 2:**
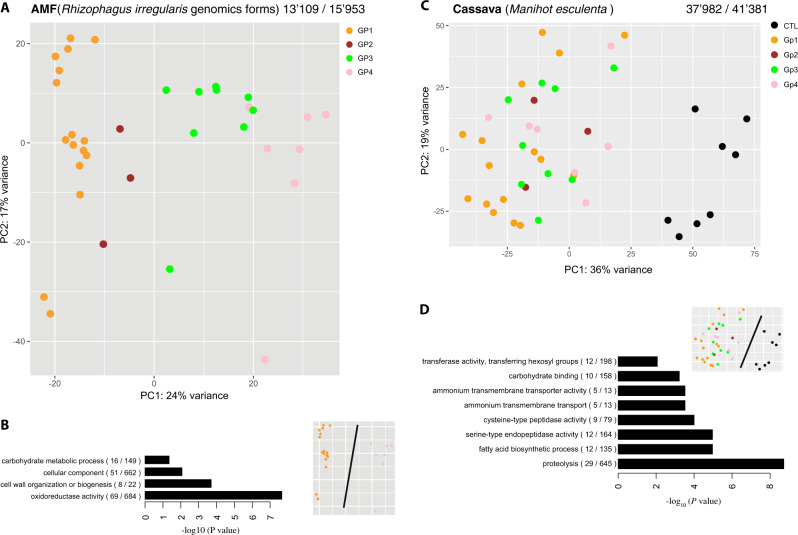
Plant and fungal gene transcription. **a** PCA of fungal gene transcription of each *R. irregularis* isolate in cassava roots. The PCA, with mock-inoculated controls (CTL) treatment is shown in Fig. S2A. Colors represent the different genetic groups of *R. irregularis*. **b** GO enrichment bar plot for the predicted function of 772 differentially transcribed fungal genes between the two most divergent fungal groups (GP1 and GP4). **c** PCA of cassava root gene transcription and colored by treatment according to the fungal genetic group. **d** GO enrichment bar plot for the function of 353 genes that were differentially transcribed between all four fungal genetic group treatments and the CTL. The numbers above each PCA (**a** and **c**) represent the number of genes found as transcripts and the total number of annotated genes in the genome assembly of the species. Following each GO function shown in the bar plots (**b** and **d**), the number of genes is shown that were differentially transcribed compared with the total number of genes in that functional category. The small PCA representation above the bar plots (**b** and **d**) depicts the treatments compared to obtain the differentially transcribed genes displayed in the bar plots.

Phylogenetically diverse *R. irregularis* isolates significantly differed in their ability to colonize cassava roots, with colonization ranging from 21.7% of the root length colonized by SAMP7 to 72.9% by DAOM197198-CZ (Fig. 1c, Data S1). Colonization was significantly associated with the phylogeny of the fungus (Fig. 1c). Mock-inoculated control roots were confirmed to not be colonized by AMF. Despite the differences in fungal colonization, there were no significant differences in plant growth, measured as root and above ground dry weight (Fig. S1). This lack in trait differences is probably due to stage at which the plants were harvested, as cassava growth was shown to vary in response to different AMF but only at late stages of growth [16, 46]. The pattern of fungal gene transcription was clearly discernible among the fungal groups (Figs. 2a and S2A, B). The fungal phylogeny based on SNPs in the genome was congruent with a phylogeny based on transcript profiles (Fig. S2C). Approximately 5.9% of the gene repertoire (772 out of 13,109 genes) of the most divergent genetic groups (GP1 and GP4) were DT. These included important functions such as cell wall organization and biogenesis (Fig. 2b). These observations confirmed the strong divergence in genetic reprogramming between these fungal groups [21] during symbiosis.

### Upregulation of the fatty acid biosynthesis pathway in cassava in response to symbiosis

A comparison of gene transcripts from mock-inoculated control roots with transcripts of all *R. irregularis* inoculated roots revealed that the genetic reprogramming of the root, as a response to inoculation, affected less than 2.5% of the cassava gene repertoire (949 out of 37,982 genes). The transcription of only 353 cassava genes (0.93%) showed a conserved transcriptional response to symbiosis consistently across all *R. irregularis* isolates (Data S2a, b). Unlike fungal gene transcripts, patterns of cassava gene transcription did not cluster into identifiable groups based on the genetic background of the fungus, but were distinct from transcripts of the mock-inoculated plants (Fig. 2c). As a test of the robustness of the transcript dataset, we searched for orthologous genes of the so-called “mycorrhizal symbiosis toolkit” necessary for fungal colonization of roots. Of eleven orthologs, known to be necessary for mycorrhiza formation in *Medicago truncatula*, ten were significantly upregulated in cassava in the presence of mycorrhizal fungi (Fig. S3A, B). GO enrichment analysis revealed that differential transcription between mycorrhizal and non-mycorrhizal plants included genes involved in known essential functions of the symbiosis such as ammonium transport and carbohydrate binding (Fig. 2d). The fatty acid pathway (GO:0006633) was proportionally the most enriched and complete in the analysis. Some of these genes were also DT between plants inoculated with the most evolutionarily divergent isolates (GP1 and GP3 + 4). For example, this included one of the key enzymes of fatty acid synthesis, an enoyl-acyl carrier reductase (Manes.08G046300.1), a carboxyltransferase alpha (Manes.09G101700.2), and trans-2,3-enoyl-reductase (Manes.07G083100.1) (Data S2c).

**Fig. 3:**
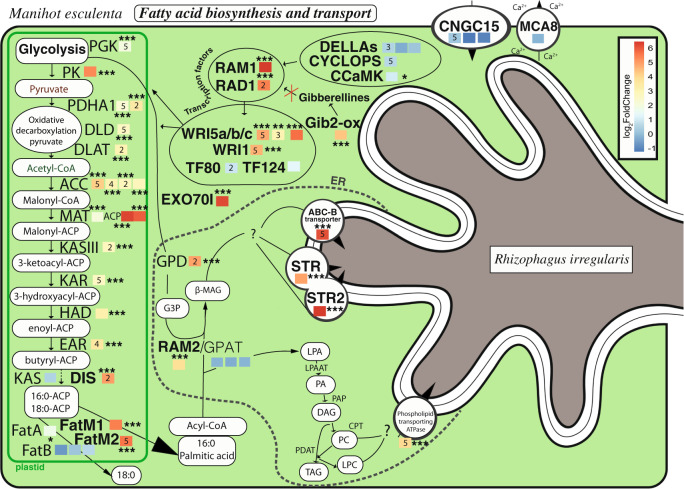
Schematic representation of gene transcriptional changes related to pathway initiation, glycolysis, fatty acid biosynthesis and transport in *M. esculenta* (cassava) in response to the AM symbiosis. Genes commonly upregulated with all fungal isolates are represented in between their products. Colored squares next to each gene represent the log_2_fold change obtained with Kallisto and DESeq2. The asterisks represent the significance of the change (* < 0.05, ** < 0.01, *** < 0.001). Numbers within each colored square represent the number of indicators of a significant phylogenetic signal (out of five indicators) between the fungal phylogeny and the transcription of a given cassava gene. Genes that were shown to play a role, or putatively play a role, in fatty acid biosynthesis are represented. For a description of each gene acronym see Data S4.

We observed the upregulation of many cassava genes comprising the whole fatty acid pathway from initiation by transcription factors, through end of glycolysis, oxidative decarboxylation of pyruvate and the fatty acid synthesis pathway (Fig. 3, Data S4). Here we show for the first time that this pathway is commonly upregulated across all genetically different AM fungal treatments in an important global food security crop.

#### Transcription factors

The transcription factor RAM1 (required for Arbuscular Mycorrhization 1) exhibited the largest fold change in transcription of all genes involved in fatty acid biosynthesis. It was proposed that RAM1 plays a central role in the activation of fatty acid biosynthesis [7]. Gibberellic acid inhibition induces the transcription of RAM1 [47]. Gibberellin-2-oxidase, that acts as a gibberellic acid inhibitor [48], was upregulated in mycorrhizal cassava (Fig. 3). This gibberellin-2-oxidase might, therefore, play an important role in the activation of RAM1 or other GRAS factors [49] (plant-specific proteins named after: GAI, RGA, and SCR). RAD1 (required for arbuscule development 1) is known to interact with RAM1 [50] and was shown to also be commonly strongly upregulated in cassava (Figs. 3 and S4A). WRI5a–c are transcription factors dependent on RAM1 [7]. Two WRI (WRI5a and c) orthologs were commonly upregulated (Figs. 3 and S4B). WRI5b upregulation was observed with one of the mapping strategies. In *Arabidopsis thaliana* these genes act at the end of the glycolysis and the mycorrhizal conserved genes WRI5 were confirmed as having a similar function in *Nicotiana benthamiana* [7, 13]. These findings confirm the tight link between these important and specific plant transcription factors with the process of fatty acid biosynthesis and their associated genes during AMF symbiosis.

#### Glycolysis and pyruvate oxidative decarboxylation

We observed common upregulation of genes involved at the end of the glycolysis and during the oxidative decarboxylation of pyruvate (Fig. 3) such as phosphoglycerate kinase (PGK) and pyruvate kinase, pyruvate dehydrogenase E1 component subunit alpha, dihydrolipoamide dehydrogenase (DLD), and dihydrolipoamide acetyltransferase component of pyruvate dehydrogenase. The genes involved in glycolysis are responsible for the production of pyruvate that will then be decarboxylated in the oxidative decarboxylation of pyruvate. At the end of this process acetyl-CoA, the precursor for fatty acid biosynthesis, will be released. In this study we showed, for the first time, that not only is the expression of the plant fatty acid biosynthesis pathway affected, but upstream plant molecular mechanisms are also impacted by AMF.

#### Fatty acid biosynthesis

Twelve genes of the fatty acid biosynthesis pathway were commonly upregulated (Fig. 3). An AM symbiosis-specific fatty acid synthesis pathway was commonly upregulated, including genes DIS (disorganized arbuscules, Fig. S4C) [15], FATM1 and 2 [51] (Fig. S4D), and RAM2 [52] (Fig. S4E). The other branch of this pathway (KASI, FATB, GPAT) was not (or poorly) upregulated. RAM2 is thought to produce β-monacylglycerol; a fatty acid exported to the outer membrane space [14]. RAM2 requires glycerol-3-phosphate. Glycerol-3-phosphate dehydrogenase gene was also commonly upregulated. Each gene and their variation for all treatments and their replicates are found in the heatmap in the supplementary information (Fig. S5A). The fatty acid biosynthesis pathway is, therefore, a commonly upregulated pathway in cassava during AMF symbiosis. As it has been suggested in model plants [7], this process is probably vital for the life of the fungus as well as probably essential for the symbiosis equilibrium in important tropical crops as well as model plant species.

#### A proposed transport route

Two ATP-binding cassette transporters (ABC-G), STR, and STR2, that were shown to be indispensable for arbuscule formation have been suggested as potential lipid transporters [14, 53]. Both were commonly highly upregulated in all fungal treatments (Fig. 3). Finally, we observed the common upregulation of a phospholipid-transport ATPase and an ABC-B transporter [54].

The common response across all fungal treatments of such a large number of genes involved in this fatty acid synthesis and export pathway in cassava, combined with the knowledge of those genes in other species and their conservation in mycorrhizal plants, indicates that the whole activation of this pathway is important for the symbiosis by producing more fatty acid as a currency of exchange with the fungi.

### Fungal genetic variation and evolutionary history drive the cassava fatty acid pathway

**Fig. 4:**
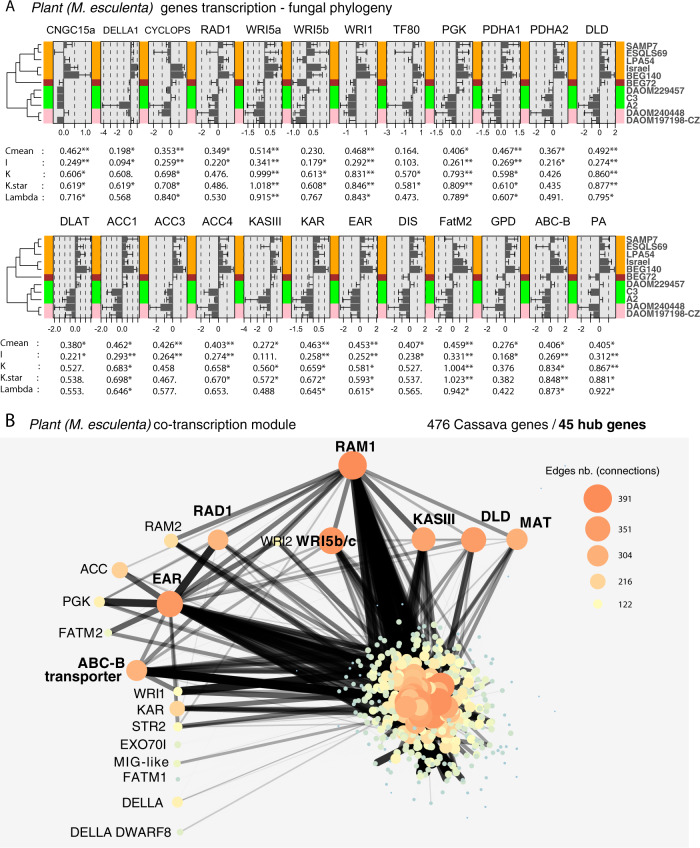
Phylogenetic signals of cassava fatty acid gene transcription significantly associated with the *R. irregularis* phylogeny and the cassava fatty acid pathway co-expression network. **a** Histograms showing the transcriptional responses of cassava genes in the different fungal treatments and represented as the difference of each treatment to the mean transcriptional response across all treatments. Bars represent ±S.E. Phylogenetic signal indicators and their significance are shown below in each histogram. Only genes that showed at least one significant indicator are displayed. Other fatty acid-related genes are shown in Fig. S5B. **b** The plant co-expression network based on one gene module comprising the largest number of genes from the fatty acid pathway. The network shows the strong co-transcription of RAM1 with several other transcription factors (WRI5c and RAD1) and fatty acid synthesis genes (DLD, KASIII, MAT, and EAR), which are also hub genes in this module (in bold). In this module, 45 were hub genes out of 476, but only the hub fatty acid-related genes are shown in bold. Edges (connections between nodes/genes) of the network are represented as a function of their weight, a measure of connection strength. Only the 5% strongest edges are represented with increase thickness and decrease transparency with connection intensity. Node size reflects the degree of connectivity, i.e., the number of connections with other nodes. More information on the network is available in Data S5a.

Having established that the fatty acid pathway is indeed upregulated in cassava in response to all the genetically different fungi, we then asked whether the quantity of upregulation of each gene is associated with identifiable patterns of genetic variation in the fungus that represent their evolutionary history. We found that variation in transcription of a striking number of cassava genes in the initiation of the fatty acid pathway, fatty acid synthesis and transport was significantly associated with the pattern of genetic variation among *R. irregularis* isolates (Fig. 4a). This included 24 genes (Fig. 4a). Other commonly DT fatty acid genes (6) and suspected fatty acid-related genes (7) not presenting this pattern can be found in supplementary material (Fig. S5B) or in Data S2a. More specifically, there was a significant relationship between patterns of genetic variation based on 15229 genome-wide SNPs in the fungi and the amount of transcription of these genes (Fig. 4a). All genes showed the same pattern of response with higher transcription in response to GP1 and a lower induction of this pathway in response to fungi in GP3 and 4. AMF cannot synthesize their own fatty acids [8]. It is, therefore, intriguing that the high inducers of the plant fatty acid pathway were significantly the lowest colonizers and that the lowest inducers of the fatty acid pathway were significantly the highest colonizers (Figs. 1 and 4a).

### Gene network and hub genes of fatty acid biosynthesis revealed by genetic differences among *R. irregularis* isolates

We built co-transcription gene modules using the variation in transcription of all the *M. esculenta* genes in all the AMF inoculated treatments. Among the 31 cassava gene modules, one gene module contained most of the fatty acid-related genes. This module contained 476 genes, of which 45 were hub genes, i.e., hub genes have the highest connectivity within the module defined as a MM above 0.9 (MM > 0.9; Data S5a). RAM1 was the hub gene with the highest MM (MM = 0.97; Fig. 4b), i.e., it had the highest connectivity to all the other genes within the module. This reveals the gene hierarchy in the plant fatty acid synthesis pathway during AMF symbiosis, with RAM1 as one of the top initiators of this cascade. Other hub genes in the module were commonly upregulated in the pathway shown in Fig. 3, e.g., WRI5c (MM = 0.95), RAD1 (MM = 0.91), DLD (MM = 0.94), MAT (MM = 0.92), KASIII (MM = 0.93), EAR (MM = 0.96), and the ABC-B transporter (MM = 0.91). Other non-hub genes occurred in this module, including RAM2, FATM1 & FATM2, WRI5a & WRI1, EXO70I, STR2, MIG-like, KAR, ACC, and PGK. In this fatty acid module several functions were enriched such as carbohydrate metabolic process, carbon utilization, inorganic phosphate transmembrane transporter activity, and phosphate ion transport. Fatty acid functions were only marginally enriched in both the hub gene list (45 genes) and the module gene list (476 genes) without correction under the term FAS complex (*P* value = 0.00012, *p*-adjusted = 0.136) (Data S5b, c). This marginally significant enrichment might be due to the low-quality annotation, where several genes known to be implicated in the fatty acid pathway (RAM1, FATM1, 2, WRI5, and other genes) are not annotated as genes with a fatty acid function. Using the same methods, we combined cassava and fungal transcripts and built new symbiosis gene modules (27 modules). The module containing RAM1 (MM = 0.95), contained 165 hub genes (Data S5d) enriched in plant genes of the fatty acid biosynthesis process (*p*-adjusted = 0.000013) and FAS complex (*p*-adjusted = 0.01, Data S5e, f). However, no *R. irregularis* genes were hub genes in the module. While this might, at first glance, suggest a full control of the plant over the fungus, there are several reasons why this may not be the case. We caution such an interpretation from these results as they would be inconsistent with theory regarding the evolution of mutualistic symbioses, where mutualism is stable when neither partner can overexploit the other.

These findings show the central role played by RAM1 during AM symbiosis, and its co-transcription with a large number of well-known genes (WRI5, RAD1) confirm a strong link of this transcription factor with several key genes of fatty acid biosynthesis that were not previously been demonstrated, such as DLD, MAT, KASIII, and EAR.

## Conclusions

These results demonstrate for the first time in the mycorrhizal symbiosis that the fatty acid induction, glycolysis, oxidative decarboxylation of pyruvate, fatty acid biosynthesis and transport are commonly upregulated during AMF symbiosis with a range of genetically diverse *R. irregularis* in cassava; one of the world’s most important food security crops. Strong transcriptional differences in response to fungal genetic variation allowed us to identify the hub genes involved in this pathway during symbiosis. More significantly, these findings are the first demonstration of the clear link between mycorrhizal fungal genetic variation and plant molecular reprogramming that reflect the evolutionary history of closely related AMF involving the production and transport of one essential currency that is fundamental to the symbiosis. To the best of our knowledge, this represents the first demonstration of an identifiable genetic basis regulating one currency of trade in a mutualistic symbiosis. While previous molecular studies have qualitatively demonstrated the switching-on of this important pathway in symbiosis, our study shows that in order to understand the regulation of the currency involved in this important mutualism, incorporate fungal genetic variation rather than using more simple experimental designs is necessary. Using only one fungal genotype and comparing it to a mock-inoculated control would not generate the variation in gene transcription levels necessary to build such a co-expression gene network. The fact that variation in plant gene regulation is linked to defined patterns of genome-wide SNPs also opens the door to finding which variation in fungal genomes leads to optimal molecular interactions in trade of resources between the mycorrhizal fungi and cassava. Such investigations could use a genome-wide association approach to identify key sites in the fungal genome. Given that cassava feeds one billion people daily, this represents a highly worthwhile pursuit as it could lead to a better understanding of how the symbiosis can be rendered more efficient in agriculture.

## Supplementary information

Supplementary Information

Data S1

Data S2

Data S3

Data S4

Data S5

## Data Availability

Data robustness was assessed in five ways, a detailed description could be found in Supplementary Note S5. Quantitative traits of the experiment, DT genes table, and co-transcribed genes table are found in Supplementary Data described in the Supplementary Note S6. The raw reads of the 47 libraries have been deposited in the NCBI under the bioproject number PRJNA428849.
