# Supplementary material for: Genetic variation and evolutionary history of a mycorrhizal fungus regulate the currency of exchange in symbiosis with the food security crop cassava
Source: ISME J. 2020 Feb 17;14(6):1333–44. doi: 10.1038/s41396-020-0606-6 (PMC7242447; doi:10.1038/s41396-020-0606-6)
Supplement: Supplementary file 1 — Supplementary Information [file 41396_2020_606_MOESM1_ESM.docx]

**Supplementary Figure S1:** AMF colonization, total root dry mass (RDM), bulking-root dry mass (BDM) and above ground dry mass (ADM) and their association with the fungal phylogeny. Different letters next to bars indicate a significant difference (*P* < 0.05). The values of the controls were taken into account for the statistical analysis of RDM, BDM and ADM (df=12), but the controls are not shown in the bar plots. Five phylogenetic signal indicators (Cmean, I, K, KStar, Lambda) are displayed with the significance of the test for an association between the quantitative trait and the phylogeny (*P* < 0.1, * *P* < 0.05, ** *P* < 0.01). Colour coding for each of the four genetic groups follows [21] (GP1: orange, GP2: brown, GP3: green, GP4: pink).

**Supplementary Figure S2: PCA of gene transcription in four *R. irregularis* genetic groups with mock-inoculated controls; fungal phylogeny including each replicate, congruence of fungal genomic- and transcriptomic-based phylogenies. Related to Figure 2 and STAR Methods.** (A) PCA showing gene transcription of *R. irregularis* isolates in the 4 genetic groups of the fungus, including all replicates and CTL. (B) Phylogeny of the *R. irregularis* isolates and replicates based on 48 SNPs observed in fungal transcripts from cassava roots. This phylogenetic tree confirms the presence of the correct isolate in the correct treatment. Only one of the replicates of the fungal treatments (A2-11) was wrongly assigned or contaminated by isolate SAMP7 or ESQLS69. This was removed from subsequent analyses. (C) Genome-based and transcriptome-based co-phylogeny of *R. irregularis* isolates of the 4 *R. irregularis* genetic groups. The genome-based phylogeny was based on ddRADseq data [1]. The fungal RNAseq clustering was based on the PCA 2 in Fig. 2A.


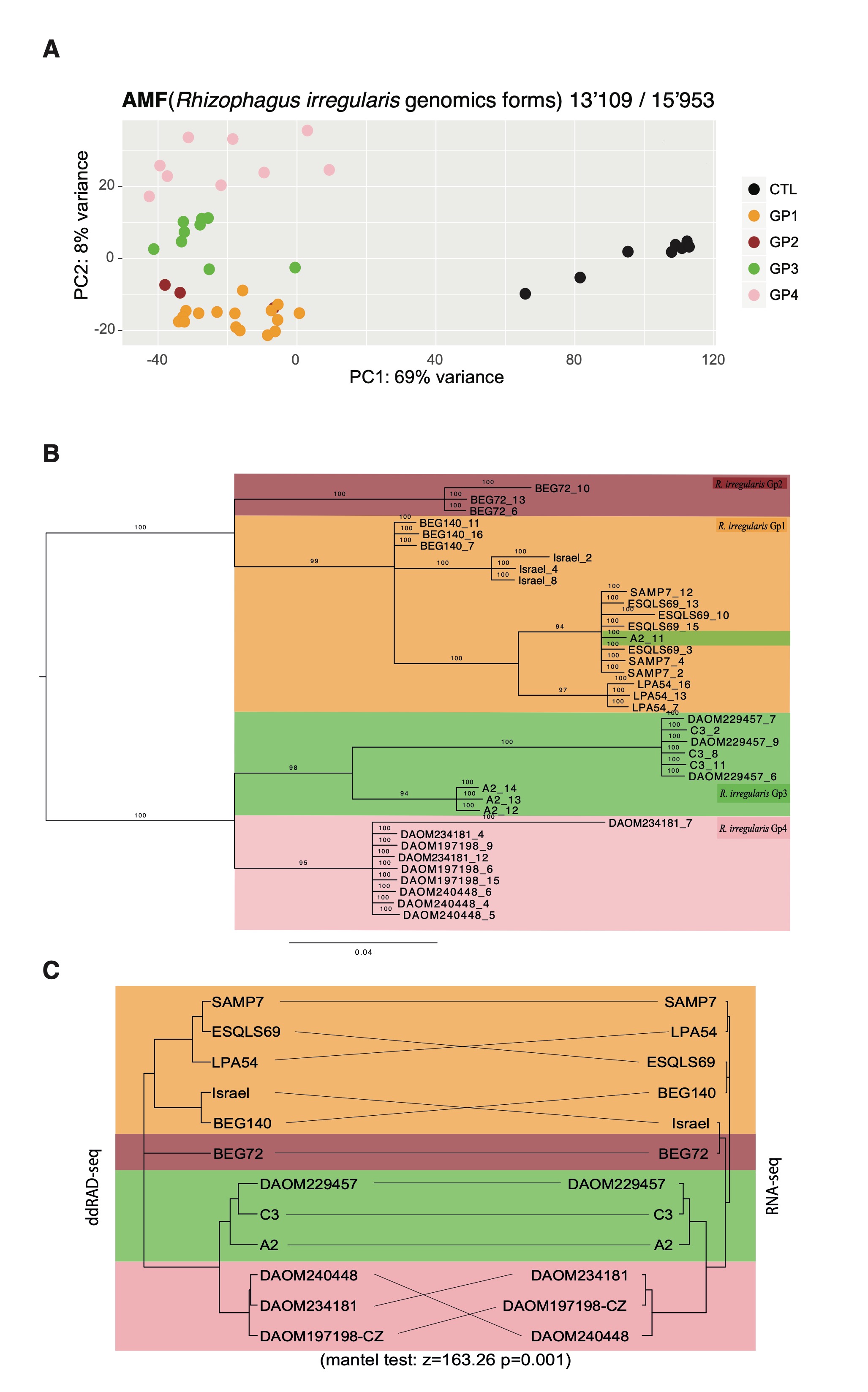


**Supplementary Figure S3: Heatmap and Volcano plot of Cassava gene transcription and change in transcription during symbiosis, with a focus on orthologs of the common “late-stage” plant genes up-regulated in the AM symbiosis, Related to Figure 2 and STAR Methods.** (A) Heatmap of 11 cassava genes known to be commonly upregulated in other model plant species during establishment of the AM symbiosis. Genes were chosen from [2]. The heatmap represents normalized transcription in all replicates of the 12 *R. irregularis* isolates. The nine mock-inoculated controls (CTL) are represented at the bottom of the figure as CTL. (B) Volcano plot representing the change in gene transcription (log_2_fold change) of all cassava genes between mock-inoculated control roots and AMF inoculated roots, according to their –log_10_ *P*-value obtained with DESeq2. Upregulated genes are represented in red and down-regulated genes are represented in blue. Genes that did not change in their transcription levels are represented in black. Cassava orthologs of plant genes commonly up-regulated during AM symbiosis of (A) are represented in green with their respective number. All these genes were found to be upregulated except Flot4 (6). Circle with annotation and gene identity in (B) depicts the 10 most up-regulated genes.

**
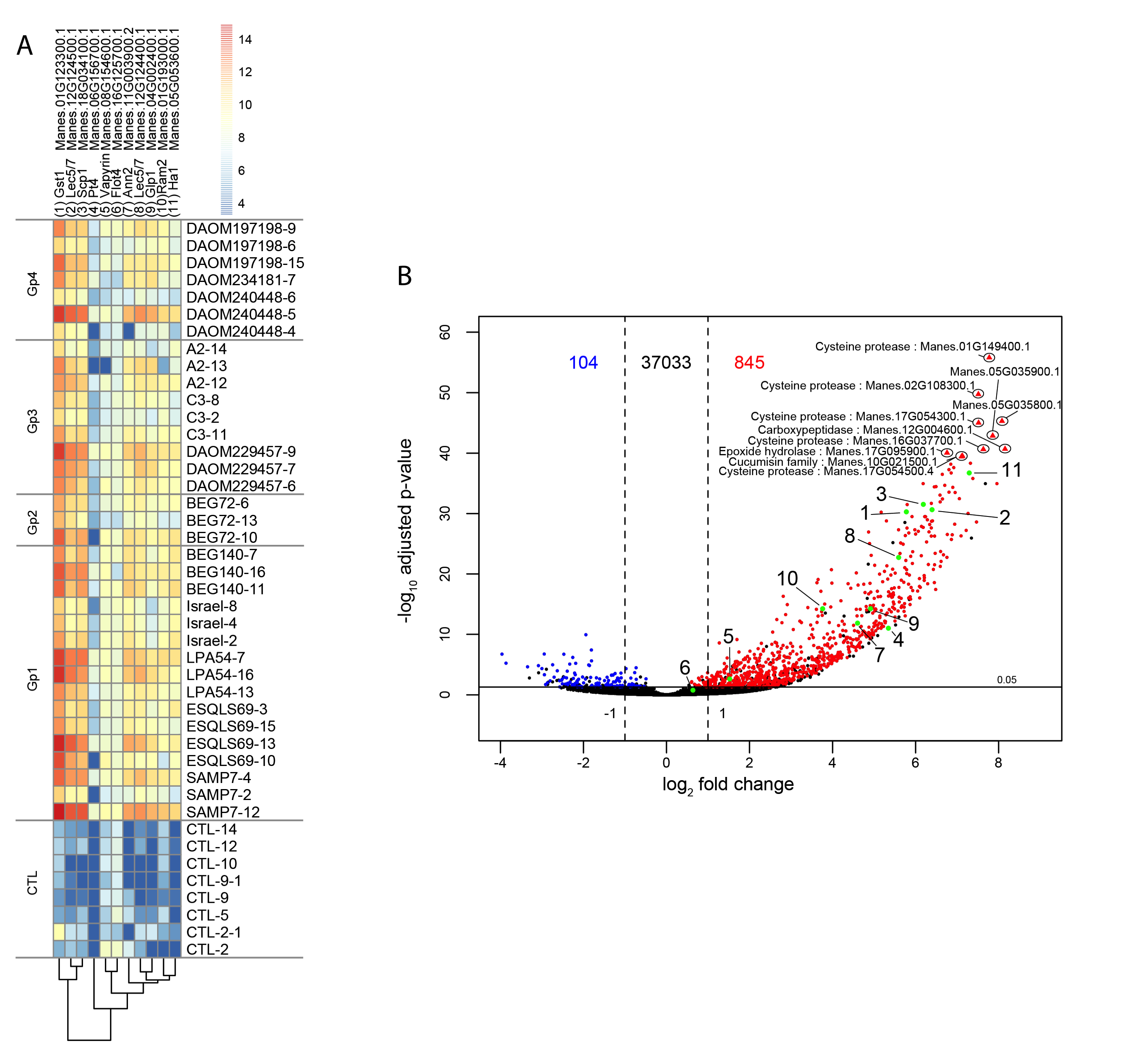
**

**Supplementary Figure S4: Cassava protein phylogenies of fatty acid biosynthesis-related genes and their change in transcription during AMF symbiosis with the 4 *R. irregularis* genetic groups. *Medicago truncatula* orthologous reference genes are included. Related to Figure 3 and STAR Methods** (A) GRAS transcription factors including MIG, RAD1, and RAM1. (B) WRINKLED. (C) DIS. (D) FATM. (E) RAM2. These genes are conserved in plants forming the AMF symbiosis.

**Supplementary Figure S5. Heatmap and tests for the relationship between fungal phylogeny and transcription of genes related to fatty acid activation, biosynthesis and transport in *M. esculenta* (Cassava) during AMF symbiosis with all isolates. Related to Figure 3, Figure 4 and Data S4**. (**A**) Heatmap of *vst* normalized transcription (Kallisto and DESeq2) of the cassava fatty acid related genes represented in Figure 3. Genes found to be commonly up-regulated in symbiosis with all four *R. irregularis* genetic groups and genes related to the fatty acid cycle. These genes include, the calcium transporters CNGC15, and ATPase MCA8, the complex of genes DELLAs-CYCLOPS-CCaMK and several DELLAs transcription factors including RAM1 and RAD1. Genes found to be commonly up-regulated are found from the end of glycolysis, during the oxidative decarboxylation of pyruvate, during fatty acid biosynthesis as well as with potential lipid transporters. The acronyms of the enzymes and genes are, Required for Arbuscular Mycorrhization 1 (RAM1), Required for Arbuscule Development (RAD1), EXO70I is required for the formation of the periarbuscular membrane, WRINKLED genes or AP2 domain proteins (WRI), phosphoglycerate kinase (PGK), pyruvate kinase (PK), pyruvate dehydrogenase alpha (PDHA1), dihydrolipoamide deshydrogenase (DLD), dihydrolipoamide acetyltransferase (DLAT), acetyl carboxylase (ACC), malonly acyl transferase (MAT), acyl-carrier-protein (ACP), beta-ketoacyl-ACP synthase III (KASIII), keto-acyl-reductase (KAR), hydroxyacyl-ACP dehydratase (HAD), enoyl-acyl carrier reductase (EAR), ketoacyl synthase or DIsorganized Arbuscules (KAS/DIS), palmitoyl-acyl carrier protein thioesterase (FATM1 & FATM2), acyl-ACP thioesterases (FATA), acyl-ACP thioesterases (FATB), reduced arbuscular mycorrhization 2 (RAM2), glycerol-3-phosphate acyltransferase (GPAT), glycerol-3-phosphate dehydrogenase (GPD), STunted Arbuscule ABC transporter (STR/STR2), phophatidic acid transporter (PA). (**B**) Gene transcriptional differences of 22 genes related to fatty acid synthesis in Fig. S4 A and whether this is associated with the fungal phylogeny with 5 phylogenetic signal indicators Cmean, I, K, K-star, Lambda.

**Supplementary Note S1:** Intraradical fungal colonization

Roots were taken from each of the 208 plants and were used for measurement of intraradical fungal colonization. This allowed us to confirm the presence of the fungus in each inoculated treatment and absence of the fungus in the CTL. Clean roots of each plant were soaked in a KOH 10% solution for 4-6 hours at 90°C, the KOH solution was changed regularly until it reached a clear colour. After completely removing the KOH solution, HCl 1% was added for 5 min and then replaced with Trypan blue for overnight straining (Trypan blue solution: 333ml of lactic acid, 333ml of ddH_2_0, 333ml of glycerol and 0.5g of Trypan blue). Trypan blue solution was then replaced with 80% lactic acid. Colonization was measured using a protocol from the International Culture Collection of (Vesicular) Arbuscular Mycorrhizal Fungi:

<https://invam.wvu.edu/methods/mycorrhizae/mycorrhizalength>. Roots were randomly spread in a Petri dish with an underlying 5mm square grid. Intersections of the roots with the grid were counted with a binocular microscope (Leica MZ 12.5). The number of roots intersecting the grid with a fungal structure divided by the total number of intersections between the roots and the grid multiplied by 100, gave the colonization rate (Data S1).

**Supplementary note S2:** RNA library preparation and sequencing

RNA was extracted from 47 root samples (Data S2a). RNA from 40 samples was extracted following a previously published protocol [3]. All solutions were prepared with DEPC (Diethyl pyrocarbonate) water. Mortar and pestles were soaked overnight in a 50% bleach and ddH_2_0 solution, rinsed with ethanol and ddH_2_0. One hundred mg of fresh roots were ground in a mortar with liquid nitrogen. The powder was transferred to 800 μl of Extraction buffer (100 mM Tris‐HCl pH 8.0, 10mM EDTA pH 8.0, 100mM LiCl, 1% SDS, 200mM β‐mercaptoethanol). Samples were homogenized and then incubated for 5 minutes at room temperature. Four hundred μl of cold Phenol:Chloroform:Isoamyl alcohol (25:24:1; PCI) was added. After mixing, samples were centrifuged at 14 000 rpm at 4°C for 15 min. The supernatant was collected and an equal amount of PCI was added. Samples were centrifuged for 10 min at 14000 rpm and 4°C. The supernatant was collected and 1 volume of isopropanol and 0.1 volume of Sodium acetate (1M, pH 5.2) was added. The samples were incubated for 10 min at room temperature and then centrifuged for 10 min at 14 000 rpm and 4°C. The pellet was washed with 500 μl ethanol 70% and dissolved in 30 μl RNase‐free water. Samples were treated with a Macherey‐Nagel Dnase kit and cleaned with the Nucleospin RNA cleanup XS kit from Macherey‐Nagel. Concentration and integrity of the RNA samples was assessed with a Nanodrop 2 000 and a Fragment Analyser^TM^ (Advanced Analytical), using the RQN integrity score.

Libraries were constructed by polyA selection of RNA. Each library was prepared with the TruSeq Stranded mRNA Sample Prep Kit® (Illumina). We selected replicate RNA samples of each treatment according to quality; selecting the ones with the highest RQN score, absence of degradation and impurities. Library concentration was assessed with Quantifluor (Promega) and quality with a Fragment Analyser^TM^ (Advanced Analytical). Libraries were sequenced using Illumina HiSeq paired-end (2 x 100nt) sequencing on Illumina Hiseq2000 platform. Forty-seven libraries were sequenced. Batches of six libraries were pooled and sequenced in eight separate lanes (Data S2a). Each lane contained at least one CTL and replicates of the different treatments were distributed, as much as possible, across lanes in order to avoid lane effects. Thus, the 3 to 4 replicates of each fungal treatment were represented into two to three lanes.

**Supplementary note S3**: Fungus genome annotations and GO enrichment analysis

A new *R. irregularis* gene annotation was generated with the following procedure. Prediction of fungal protein coding genes was performed with the *ab initio* gene prediction tool Augustus, based on a hidden Markov model [4]. Augustus was trained with all *Aspergillus* proteins manually curated by Swissprot and aligned on the N6 *R. irregularis* genome with BLAT to define the gene structure [5]. The structure of the genes was used to train and optimize Augustus following Augustus instructions:

(<http://www.molecularevolution.org/molevolfiles/exercises/augustus/training.html>).

We generated hints for Augustus predictions from DAOM197198 RNA-seq data using STAR aligner [6] and cufflinks [7]. Augustus predictions with hints were performed on the repeat-masked N6 genome using the trained parameters for the species. Proteins were blasted against UniProtKB fungal protein database ([www.uniprot.org](http://www.uniprot.org/)); the blast hits were then used in Blast2GO [8] in order to obtain GO terms.

**Supplementary note S4:** Differential transcription in *M. esculenta* and *R. irregularis*

*M. esculenta* transcript counts were analysed with the DESeq function, which includes a normalization for library size. First, a global comparison including controls compared to all fungal inoculation treatments was performed in order to obtain *M. esculenta* transcripts DT during symbiosis with AMF. The transcripts significantly DT, in the comparison CTL *versus* inoculated, with both mapping strategies were considered as core transcripts of the *M. esculenta*–*R. irregularis* symbiosis. These transcripts were kept for gene ontology (GO) enrichment analysis and volcano plot visualization of the most highly DT transcripts (Figure S2B).

Second, in order to obtain a more precise picture of core *M. esculenta* transcription during symbiosis with *R. irregularis* and commonly DT in each *R. irregularis* genetic group, we performed the following comparisons; i) CTL vs Gp1, ii) CTL vs Gp2, iii) CTL vs Gp3, iiii) CTL vs Gp4. The intersect of each of these four lists of DT transcripts produced a second list of core genes, but this time each transcript was significantly DT in each genetic group compared to CTL plants. This was repeated after removing the seven libraries that were extracted with the second RNA extraction method using the Maxwell^TM^ robot and with both mapping strategies.

Finally, we compared Cassava transcription resulting from inoculation with the two main phylogenetically diverged branches of *R. irregularis* (GP1 vs GP3+GP4). As GP2 contained only one isolate and represented a distant branch of GP1, we did not include this isolate in this comparison. These lists of commonly DT genes were used for GO enrichment analysis. In *M. esculenta* analyses, four libraries were removed: CTL8 due to the small number of reads. This sample was also considered contaminated by fungal reads. DAOM234181-12 and DAOM234181-4 due to their poor quality. A2-11 due to a contamination with another fungus (see Figure S2B).

*R. irregularis* count tables were also analysed with the DESeq2 function. The CTL treatments were removed, as they did not contain *R. irregularis* reads. Comparisons were performed among the four genetic groups with all pairwise permutations. Thus, GP1 was compared to GP2, GP3 and GP4. GP2 was compared to GP3 and GP4. GP3 was compared to GP4. The six lists of DE genes were used for GO enrichment analysis. Here we only report the comparison with the two most phylogenetically diverged *R. irregularis* groups, namely GP1 and GP4.

**Supplementary note S5:** Robustness of the data

The robustness RNA transcript data was assessed in fives ways to check that mock-inoculated treatments were free of *R. irregularis* transcripts and that the inoculated plants were truly mycorrhizal and colonized by the expected fungus. First, all CTL samples were only retained if AMF colonization was zero. Similarly, fungal inoculated treatments were only kept if colonization was different from zero. Second, colonization was also assessed as a percentage of reads mapping to the *R. irregularis* genome compared to the mapped reads of the *M. esculenta* genome. The percentage of *R. irregularis* reads compared to *M. esculenta* reads was used to discard CTL plants that were considered as contaminated if reaching over 1%. Only CTL (replicate 8) exhibited a fungal read percentage higher than 1% (1.15%) and this was removed from the analysis. Third, in combination with the read percentage, the PCA of *R. irregularis* normalized counts (including reads from the CTL treatment) was used to visualize and discard any potentially contaminated controls. We also removed libraries DAOM234181-12 and DAOM234181-4 due to low sequence quality*.* Fourth, we used FreeBayes [9] in order to call SNPs and build a phylogeny [1] of the *R. irregularis* mapped reads to confirm the presence of the correct *R. irregularis* isolate. Only one of the treatments was found to be contaminated or wrongly assigned. The treatment with isolate A2 (A2-11) appeared to have been contaminated with a fungus from GP1 (either SAMP7 or ESLQS69; Figure S2B). This treatment was removed from all analyses. Fifth, we searched in the main results for orthologs of 11 well-known plant genes (Gst1, Lec5, Scp1, Pt4, Vapyrin, Flot4, Ann2, Lec7, Glp1, Ram2, Ha1) commonly differentially transcribed during AMF symbiosis. Genes were chosen from [2]. We only selected genes for which orthologs in cassava could be found and which are not typically expressed in the very early stages of symbiosis formation. Ten of the 11 genes were found to be clearly commonly differentially transcribed, confirming that plants in all the fungal treatments were colonized (Figure S3A and B). Removing the 7 samples that were extracted with the second extraction method and reanalysing the data did not change the conclusions on the manuscript. Indeed, from the 353 genes commonly DT with all the *R. irregularis* treatments, 310 are found commonly DT after removing the 7 libraries. In these 310 genes, the enrichment for genes DT of fatty acid biosynthesis was also significant.

**Supplementary note S6:** Data availability

Quantitative traits (ADM, RDM, BDM, Colonisation) of the 208 cassava plants inoculated with the 12 treatments and the control and details of the block design are available in Data S1.

A supplementary table including the 353 cassava genes commonly DT between CTL and each of the treatments of the genetic groups (GP1, GP2, GP3, GP4) is found in Data S2a. This table includes the gene identity, its annotation, its common gene name, the p-values for the fives tests for phylogenetic conservatism (Cmean, I, K, K.star, Lambda), the log_2_Fold Changes obtained with DESeq2 and their associated adjusted p-values for each genetic group. A similar table with the 949 genes differentially transcribed (DT) between CTL and AMF treated cassava is found in Data S2b. Twenty-five cassava genes are also reported as being differentially transcribed between roots inoculated with the two main branches of the *R. irregularis* phylogeny, GP1 and GP3+GP5 (Data S2c). Data S4 lists the 53 plants genes related to fatty acid biosynthesis and transport that were found to be DT, or that are known to have an impact on plant fatty acid biosynthesis. Data S5, lists all the 476 gene and the 45 hub genes of the Cyan module (476 genes); the module that contained RAM1. This table includes the *M. esculenta* code name, common gene name, the annotation, module membership (MM) and module membership p-value. This data includes the 1349 genes present in the symbiosis gene module and the 165 hub genes. General information concerning the 47 libraries is found in Data S3. The mapping information is also provided in Data S3. The raw reads of the 47 libraries have been deposited in the NCBI under the bioproject number PRJNA428849.

**Data S1**. Experimental design with block number, cassava quantitative growth traits and colonization, related to Figure 1.

**Data S2 a.c**. Lists of differentially transcribed Cassava genes for CTL vs GP1,2,3,4 (353) , CTL vs AMF (949) and between GP1 and GP3,4 (25), Related to Figure 2.

**Data S3 a-e.** Sequencing information and mapping summaries for Kallisto and 2pass STAR, Related to Supplemental Information.

**Data S4**. Fifty-three fatty acid biosynthesis genes and related genes with complementary information for Fig. 3. Related to Figure 3.

**Data S5 a-f**. List of gene belonging to the Cassava RAM1 gene module (Cyan, 476 genes, 45 hub genes) and enrichment of hub genes (45) and all genes (476). List of genes of the mixed plant and fungal RAM1 gene module (Yellow, 1349 genes, 165 hub genes) and enrichment of hub genes (165) and all genes (1349), Related to Figure 4.

**References**

1 Savary R, Masclaux FG, Wyss T, Droh G, Corella JC, Machado AP *et al* (2018). A population genomics approach shows widespread geographical distribution of cryptic genomic forms of the symbiotic fungus *Rhizophagus irregularis*. *ISME Journal* **12:** 17-30.

2 Hogekamp C, Kuster H (2013). A roadmap of cell-type specific gene expression during sequential stages of the arbuscular mycorrhiza symbiosis. *Bmc Genomics* **14**.

3 Das A, Saha D, Mondal TK (2013). An optimized method for extraction of RNA from tea roots for functional genomics analysis. *Indian Journal of Biotechnology* **12:** 129-132.

4 Stanke M, Steinkamp R, Waack S, Morgenstern B (2004). AUGUSTUS: a web server for gene finding in eukaryotes. *Nucleic Acids Research* **32:** W309-W312.

5 Kent WJ (2002). BLAT - The BLAST-like alignment tool. *Genome Research* **12:** 656-664.

6 Dobin A, Davis CA, Schlesinger F, Drenkow J, Zaleski C, Jha S *et al* (2013). STAR: ultrafast universal RNA-seq aligner. *Bioinformatics* **29:** 15-21.

7 Trapnell C, Williams BA, Pertea G, Mortazavi A, Kwan G, van Baren MJ *et al* (2010). Transcript assembly and quantification by RNA-Seq reveals unannotated transcripts and isoform switching during cell differentiation. *Nature Biotechnology* **28:** 511-U174.

8 Conesa A, Gotz S, Garcia-Gomez JM, Terol J, Talon M, Robles M (2005). Blast2GO: a universal tool for annotation, visualization and analysis in functional genomics research. *Bioinformatics* **21:** 3674-3676.

9 Garrison, E. & Marth, G (2012). Haplotype-based variant detection from short-read sequencing. arXiv preprint arXiv:1207.3907 [q-bio.GN].
